# Tissue-Mimicking Materials for Breast Ultrasound Elastography Phantoms: A Systematic Review

**DOI:** 10.3390/polym17040521

**Published:** 2025-02-17

**Authors:** Wadhhah Aldehani, Adel Jawali, Sarah Louise Savaridas, Zhihong Huang, Luigi Manfredi

**Affiliations:** 1 Division of Respiratory Medicine and Gastroenterology, School of Medicine, University of Dundee, Dundee DD1 4HN, UK; a.jawli@dundee.ac.uk; 2 Division of Population Health and Genomics, School of Medicine, University of Dundee, Dundee DD1 9SY, UK; s.savaridas@dundee.ac.uk; 3 School of Physics and Engineering Technology, University of York, York YO10 5DD, UK; zhihong.huang@york.ac.uk

**Keywords:** breast ultrasound, phantom, tissue-mimicking materials, elastography

## Abstract

Breast ultrasound elastography phantoms are valued for their ability to mimic human tissue, enabling calibration for quality assurance and testing of imaging systems. Phantoms may facilitate the development and evaluation of ultrasound techniques by accurately simulating the properties of breasts. However, selecting appropriate tissue-mimicking materials for realistic and accurate ultrasound exams is crucial to ensure the ultrasound system responds similarly to real breast tissue. We conducted a systematic review of the PubMed, Scopes, Embase, and Web of Sciences databases, identifying 928 articles in the initial search, of which 19 were selected for further evaluation based on our inclusion criteria. The chosen article focused on tissue-mimicking materials in breast ultrasound elastography phantom fabrication, providing detailed information on the fabrication process, the materials used, and ultrasound and elastography validation of phantoms. The phantoms fabricated from Polyvinyl Chloride Plastisol, silicon, and paraffin were best suited for mimicking breast, fatty, glandular, and parenchyma tissues. Adding scatterers to these materials facilitates accurate fatty and glandular breast tissue simulations, making them ideal for ultrasound quality assurance and elastography training. Future research should focus on developing more realistic phantoms for advanced medical training, improving the practice of difficult procedures, enhancing breast cancer detection research, and providing tailored tissue characteristics.

## 1. Introduction

Ultrasound is frequently used to characterize breast lesions identified clinically or mammographically. Ultrasound is also the primary imaging technique used to examine younger women due to the desire to avoid ionizing radiation and the likelihood that mammographic sensitivity is lowered by dense breast tissue [1].

Though ultrasound is an accurate technique with an average sensitivity and specificity for detecting breast malignancy of 87% and 88.4%, respectively [2], false negative results may occur when the lesion is isoechoic to breast tissue, is hidden by other breast structures, or is too small or deep to be identified. According to the Breast Imaging Reporting Data System (BI-RADS) criteria defined by the American College of Radiology (ACR) [3], various characteristics of the lesions must be evaluated [4]. The BI-RADS classifies lesions according to the likelihood of malignancy: BI-RADS 1 represents normal findings, BI-RADS 2 indicates benign findings, BI-RADS 3 represents indeterminate findings, BI-RADS 4 indicates suspicions of malignancy, and BI-RADS 5 represents findings with a high likelihood of malignancy. In clinical practice, a biopsy is recommended for lesions classified as BI-RADS 4 and above, with cancer detection rates between 10% and 30% [5]. Whilst it is essential to identify all instances in which cancer is present successfully, many benign lesions are biopsied as a result, causing discomfort and increasing costs for patients [6]. Thus, ultrasound elastography was developed to overcome these limitations and more accurately characterize breast lesions [7].

The clinical value of ultrasound elastography depends on the accuracy and reproducibility of the imaging process [8]. For breast ultrasound elastography systems, tissue-mimicking phantoms that accurately reproduce the mechanical properties of human breast tissue are invaluable [9].

Tissue-mimicking phantoms can be used to calibrate systems [10], assess image quality [11], train operators [12], and evaluate the effectiveness of systems [13]. Tissue-mimicking phantoms also offer various benefits to researchers and clinicians, allowing them to simulate real clinical scenarios and assess the performance of elastography systems under controlled conditions [14]. Therefore, the choice of materials for these phantoms plays an important role in determining the accuracy and reproducibility of the results obtained through ultrasound and elastography [15]. Using realistic tissue-mimicking materials to create phantoms allows educators to provide students with hands-on experiences that are closely related to those encountered in clinical practice [9]. As a result of these experiences, healthcare practitioners will be better equipped to interpret elastography results accurately, with concomitant improvements in patient care and diagnostic accuracy [16].

This systematic review aimed to evaluate the most durable, cost-effective, and easily fabricated materials for existing breast ultrasound phantoms used for diagnostic and research purposes.

## 2. Materials and Methods

This systematic review was registered on PROSPERO (the International Prospective Register of Systematic Reviews, #CRD42023444047).

We performed a structured literature search of PubMed, Scopus, Embase, and the Web of Science databases, using keywords related to breast ultrasound elastography phantom fabrication in order to identify relevant publications. All searches were conducted on 17 August 2023. The search terms included breast ultrasound, phantoms, tissue-mimicking materials, and elastography.

### Article Selections

To eliminate any potential bias during the study screening process, we used the web-based tool Rayyan. The inclusion and exclusion criteria are shown in Table 1. The screening was carried out independently and randomly by W.A. and A.J. In a disagreement regarding study eligibility, Z.H. was consulted to make the final decision. Once all discrepancies were resolved, authors W.A. and A.J. worked to obtain the full texts of studies that were either included or deemed potentially eligible before reviewing them in depth to determine whether they met the inclusion criteria. Similarly, any disagreements were settled through a discussion with Z.H. Additional articles were identified through citation searches, and their full texts were obtained and reviewed according to the same procedure.

**Figure 1:**
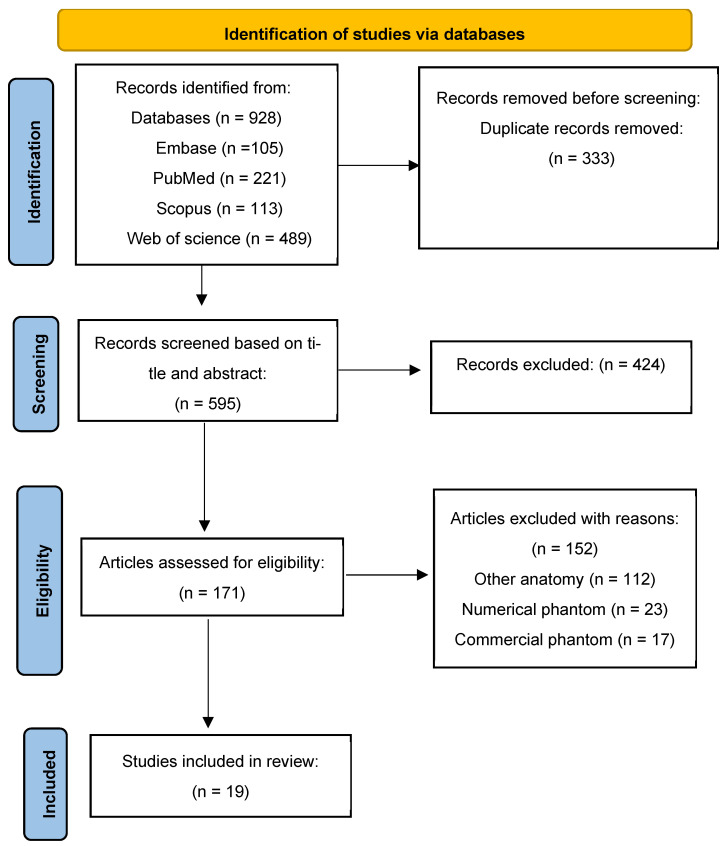
PRISMA four-phase flow diagram outlining the identification and selection procedures for the studies.

Figure 1 illustrates the different phases of a systematic review based on PRISMA recommendations for reporting systematic reviews and meta-analyses [17].

## 3. Results

A flowchart representing the study selection process is shown in Figure 1. A total of 928 articles were identified via PubMed (n = 221), Scopus (n = 113), Web of Science (n = 489), and Embase (n = 105). Duplicate articles were removed (comprising 333 articles), and a title and abstract screening was performed by A.W. and J.A., resulting in the exclusion of another 424 articles. Of the 171 full-text articles assessed for eligibility, 19 full-text articles were included in this systematic review. The identified studies provide unique contributions to the development, characteristics, and application of tissue-mimicking materials (TMMs) in teaching this procedure. The details of the included studies are summarized in Table 2, which highlights the variety of materials and physical properties that contribute to enhancing the accuracy of phantoms used for training and diagnostic purposes.

All of the included studies focused on developing breast phantoms capable of mimicking the properties of human breast tissue for use in medical imaging and training. The primary focus was on fabricating phantoms with realistic mechanical and acoustic properties that can be used to improve diagnostic accuracy and provide high-quality training for medical practitioners. For ease of understanding, the studies have been categorized by material: those made with biopolymers and those made with chemically synthesized polymers.

### 3.1. Biopolymers

Regarding biopolymers, agar-based phantoms are a prominent choice, with various studies exploring the effects of different additives on their properties [20,21,24,28]. While these phantoms use agar and degassed water as a base, incorporating substances such as olive oil, sulfate oil, detergents, evaporated milk, and glycerol introduces various mechanical and acoustic characteristics and can significantly affect the phantoms’ ability to simulate tissue properties by altering their texture and composition. For instance, adding oils tends to modify echogenicity, while substances such as glycerol can alter elasticity. These alterations highlight agar’s adaptability as a base material and emphasize the importance of tailored additive selection to achieve desired phantom characteristics. In a study by Cannon, the researchers successfully developed and characterized agar-based TMMs capable of replicating the acoustic properties of various breast tissues for high-frequency ultrasound applications. Such materials are suitable for use in quality assurance and anthropomorphic phantoms, offering more accurate and clinically relevant means of testing and training when conducting ultrasound imaging of breasts [37]. Meanwhile, Browne used the same fabrication materials and processes across multiple studies. In their initial study in 2017, their primary aim was to develop and evaluate a magnetic resonance imaging (MRI)-compatible breast rib phantom for assessing ultrasonic thermal exposures [24]. In 2019, their focus shifted to assessing the effectiveness of novel anthropomorphic breast ultrasound phantoms in improving radiology resident education [27]. Meanwhile, Ng and Lin used agar-based phantoms to investigate the tunability of acoustic and mechanical behaviors in breast tissue-mimicking materials for realistic ultrasound imaging and needle insertion feedback [28]. Ruvio used an IEC agar-based phantom to develop multimodal breast phantoms for use in various imaging techniques, including microwave, ultrasound, mammography, MRI, and computed tomography (CT) [30], while Schmidt used simple agar and degassed water to investigate whether a training program on breast ultrasound skills, including core-needle biopsies using a phantom, improved medical knowledge and learning satisfaction among undergraduate students [33]. In another study, Manickam added graphite powder to their breast elastography training phantom, which they then developed using various elastography systems and numerical simulations [21]. Altun used phantoms containing gelatine, glycerol, and degassed water to measure the acoustic impedance of tissue-mimicking materials via scanning acoustic microscopy [32]. In a study by Chun-Yen et al., the researchers used agar and evaporated milk to create and characterize an ultrasound and CT phantom for a non-invasive ultrasound thermometry calibration [20].

### 3.2. Chemically Synthesized Polymers

In total, 10 studies used chemically synthesized polymers. Vieira et al. investigated the use of paraffin gel waxes as novel soft tissue-mimicking materials for ultrasound-guided breast biopsy training [19]. Suzuki et al. used a combination of SEBS (styrene ethylene/butylene styrene) and paraffin oil to develop and evaluate an oil gel-based phantom for measuring the quantitative accuracy of the speed of sound in ultrasound computed tomography [29]. Furthermore, in a study by Kashif et al., silicone-based phantoms were used to create breast phantoms and evaluate their effectiveness in elastography imaging [18]. Ustbas et al. created and characterized composite materials that simulate breast tissue for use in ultrasonography training phantoms [26]. De Carvalho used polyvinyl chloride plastisol (PVCP) as a tissue-mimicking base material for ultrasound imaging in order to describe the design and manufacture of breast lesions [22], while Jeong used the same materials to demonstrate the phantom’s applicability to photoacoustic imaging studies [23]. De Matheo used PVCP TMMs to create breast phantoms with structures similar to human tissues, which were suitable for various imaging techniques [25]. Meanwhile, Chatelin investigated phantoms’ utility in magnetic resonance (MR) and ultrasound elastography [31]. Leonov developed a PVCP-based anatomical breast phantom featuring lesions of varying shapes, elasticities, and echogenicities, which could be used as a teaching aid for students learning to perform ultrasound examinations [35].

The development of phantoms in biomedical research serves multiple critical purposes, each aimed at enhancing these phantoms to facilitate their use in medical imaging, training, and research. A significant focus has been on assessing and training medical professionals, particularly in elastography and ultrasound imaging. Brown [24], De Matheo [25], Ustbas [26], Ng and Lin [28], Ruvio [30], Schmidt [33], and Leonov [35], among others, have demonstrated how phantoms can improve practitioners’ diagnostic accuracy by providing controlled, repeatable environments for skill training. For instance, phantoms with inclusions mimicking lesions of varying stiffness have been widely used in training programs, enabling clinicians to better differentiate between benign and malignant tissues under ultrasound [27]. Phantoms are effective for both training and research due to their material stability and realism. Gelatine and agar-based hydrogels are among the most frequently used materials due to their biocompatibility, tunable mechanical properties, and acoustic similarities to human tissues. When combined with additives such as graphite or glycerol, these materials become more durable while retaining their ability to mimic the mechanical and acoustic characteristics of soft tissues. Success in this domain is often measured by the phantom’s longevity under repeated use and its ability to maintain its mechanical and acoustic properties over time. Tailoring phantoms’ properties is key to realistic simulations, allowing experts to bridge the gap between experimental setups and clinical scenarios. Advanced phantoms are designed to replicate specific pathological features, such as fibroadenomas or malignancies, to enhance their relevance for targeted applications. PVC-based phantoms, for example, have attracted a great deal of attention due to their ability to simulate both fatty and glandular breast tissues, as demonstrated by Leonov. These tailored models are beneficial not only in terms of realism but also in terms of their ability to provide a robust platform for validating new imaging techniques; thus, they are valuable tools for medical research and education.

## 4. Discussion

Based on the studies identified in this systematic review, tissue-mimicking materials for breast ultrasound elastography phantoms have facilitated significant advancements and challenges in medical training.

### 4.1. Comparison of Materials and Their Properties

#### 4.1.1. Biopolymer-Based Properties

Agar and gelatine contain more than 80% water by mass and remain stable at temperatures up to 90 °C. As a base material, agar modifies phantoms’ structural properties, such as the elastic modulus. Based on all the included studies, we concluded that while agar is a suitable material that may be used to fabricate different human tissues, it is disadvantageous due to its lack of durability and its susceptibility to fungi and bacterial growth [21].

The training program described in the study by Schmidt significantly enhanced the theoretical knowledge of undergraduate students, who were allowed to practice the skills taught during the course. Hands-on training using a phantom proved effective, especially when performing core needle biopsies. This method was cost-effective, enhanced students’ confidence, and reduced their error rates. Cannon developed and characterized novel tissue-mimicking materials for high-frequency breast ultrasound phantoms and determined that TMMs can be used in quality assurance (QA) and anthropomorphic phantoms, resulting in improved experimental measurements of QA parameters and a better correlation with clinical outcomes. In Browne’s study, conducted in 2019—a continuation of their previous work from 2017—phantoms were used to simulate the morphological and sonographic characteristics of breast tissue during a training workshop [27]. Following the training session, lesion detection and characterization scores increased by 17 and 14%, respectively, with a *p*-value of less than 0.003, indicating that the phantoms provided an effective and realistic baseline for residents to practice their skills. The study concluded that simulation training workshops can significantly benefit radiology residents’ development of breast ultrasound imaging skills and may bolster their confidence. Ng and Lin demonstrated that tissue-mimicking materials can be customized to mimic human breast tissues’ acoustics and mechanics. Their study examined the need for realistic training phantoms to improve how ultrasound-guided breast procedures are performed by radiology residents while using the International Electromechanical Commission (IEC) agar-based TMMs and their weight percentages [38,39]. This level of tunability allows for the creation of realistic training phantoms that can enhance the realism of ultrasound imaging and needle insertion feedback, with beneficial effects on the education of radiology residents. A similar concept was developed by Ruvio, who also created phantoms that mimic mechanical and electromagnetic characteristics, enabling more comprehensive training and assessment. Indeed, Ruvio et al. investigated the development and evaluation of multimodal anthropomorphic breast phantoms designed for assessing various imaging techniques, including ultrasound, MRI, mammography, and CT. The phantoms effectively simulated the heterogeneity, anatomy, morphology, mechanical properties, and dielectric properties of real breast tissues.

Altun used a mixture of gelatine and additives to fabricate human tissues like breasts. The acoustic impedance values for breast TMM samples ranged from 1.373 to 1.707 MRayl. TMMs were found to have acoustic impedance values close to those of actual human tissues, suggesting they may be useful for ultrasound imaging and diagnostic procedures [32].

#### 4.1.2. Chemically Synthesized Materials

Kashif used breast phantoms made of silicone, which mimicked the elastic and damping properties of various human breast tissues with storage modulus (E′) values ranging from 2 to 570 kPa. Kashif successfully developed breast phantoms that mimicked skin, adipose, cancerous tumors, and pectoral muscles and tested them using Digital Image Elasto-Tomography (DIET) and Magnetic Resonance Elastography (MRE), demonstrating a significant contrast between healthy tissues and cancerous tissues [18]. Ustbas developed silicone-based phantoms for sonography training. When tested, polydimethylsiloxane (PDMS) achieved an SOS of 1290 m/s and 12.99 dB/cm attenuation.

PDMS phantoms are deemed realistic, durable, accessible, and affordable, making them suitable for ultrasound training, needle biopsy practice, and self-exam training. However, PDMS phantoms have reduced values compared to the acoustic properties of human breast tissue, which has a sound velocity of 1430–1570 m/s and an attenuation coefficient of 9.5–12.6 dB/cm [26]. Improving the acoustic accuracy of silicone-based materials requires careful modification of their composition to better match the properties of human tissues. One approach incorporates additives such as graphite powder, silica nanoparticles, or polymer blends to adjust acoustic impedance and attenuation, thereby reducing wave distortion and improving ultrasound imaging quality [40]. Additionally, modifying the crosslinking density and introducing microbubbles or scattering agents can help fine-tune both mechanical and acoustic properties, bringing them closer to those of soft tissues. While these modifications can enhance the performance of silicone-based phantoms, achieving a perfect match remains challenging due to the inherent differences in material structure. Continued research into optimizing formulations and fabrication techniques is essential to improving the reliability and clinical relevance of silicone-based phantoms for elastography applications [41].

#### 4.1.3. Polyvinyl Chloride Plastisol-Based Materials

Polyvinyl chloride (PVC)-based materials have been used for phantom fabrication in a variety of studies. PVCP was first introduced as a tissue-mimicking material for breast ultrasound elastography phantom fabrication by De Carvalho in 2015. A realistic breast tissue phantom was created for use in ultrasound imaging, specifically for training and system optimization. This material was selected because of its stability, durability, non-toxicity, low cost, and ease of handling. An in-depth analysis of the acoustic properties of the phantoms was conducted, and the phantoms successfully replicated the image characteristics of both fatty breast tissue and typical breast lesions [22]. Meanwhile, Jeong introduced a method for reducing image noise and improving ultrasound imaging quality by eliminating air bubbles during the fabrication process. The PVCP phantom demonstrated a stable performance over time, providing high-resolution photoacoustic signals.

In 2023, Hariyanto evaluated PVC powder combined with various plasticizer concentrations as a tissue-mimicking phantom material for dual-modality imaging, specifically mammography and ultrasound. The physical properties of these phantoms, such as their density, speed of sound (SoS), acoustic impedance (Z), and acoustic attenuation, were considered in relation to human breast tissues. The SoS varied from 1536.6 to 2021.2 m/s, and the acoustic impedance values ranged from 1.47 to 2.39 (10⁶ kg/m^2^/s), closely resembling human tissue values. In another study, a phantom was created using PVC in combination with dioctyl terephthalate (DOP) and other additives [36]. Compared with traditional tissue-mimicking materials such as gelatine, agar, and polyvinyl alcohol (PVA), the inherent properties of PVC, such as resistance to bacteria, stability, and reusability, provide a distinct advantage. However, these traditional materials are limited due to their propensity toward water evaporation and bacterial growth, which reduces their longevity and reusability. The studies concluded that PVCP phantoms are reliable preclinical and clinical imaging research tools, offering a stable, cost-effective, and realistic alternative to traditional tissue-mimicking materials. Indeed, PVCP phantoms can potentially enhance the development, testing, and training processes within ultrasound imaging, providing a reliable and reproducible means of simulating a wide range of breast tissue conditions.

### 4.2. Clinical and Training Applications

In medical imaging, phantoms provide a controlled and consistent training and equipment calibration environment. Phantoms with known properties serve as benchmarks for evaluating imaging systems and techniques, ensuring consistency and accuracy in clinical practice [42]. The stability and durability of tissue-mimicking materials are crucial for their effectiveness in breast ultrasound training and standardization [16]. While environmentally friendly and biocompatible, biopolymers tend to degrade more rapidly, limiting their long-term usability [43]. In contrast, chemically synthesized polymers offer greater longevity, retaining their mechanical and acoustic properties over time. This distinction is particularly important for training and quality assurance, where consistent imaging conditions are essential [23]. A material that degrades within a week would require frequent replacement, introducing variability and increasing costs. While biopolymers have valuable applications, chemically synthesized polymers provide a more practical and reliable option for sustained use in breast ultrasound phantoms. PVC provides practitioners with a standardized training tool for applying the correct pressure during elastography exams, reducing the presence of artifacts and improving diagnostic accuracy. PVC also helps to ensure that shear wave elastography measurements remain consistent across different vendors and equipment, promoting accurate research findings and clinical guidelines.

### 4.3. Review Challenges and Limitations

Although the development of breast phantoms has progressed significantly, several challenges have yet to be addressed. Comparing materials across studies was challenging, as not all papers provided detailed discussions of their properties, which limited the comparison to their acoustic properties only. Biopolymer-based materials are limited by water evaporation, bacteria growth, and lack of durability, while silicone-based materials, though durable, fall short in terms of their acoustic accuracy compared to human tissues. PVCP phantoms address many of these issues but require further improvement in fabrication techniques to ensure widespread adoption. Additionally, head-to-head comparisons of TMMs under controlled experimental conditions are needed for comprehensive performance validation. Future efforts should focus on developing materials that balance accuracy, reproducibility, and cost-effectiveness to maximize the potential of TMMs. PVCP phantoms address many of these issues, but further improvements in fabrication techniques are required to ensure their widespread adoption. Simplified fabrication methods and multimodal integration are important for advancing medical imaging training and diagnostics, and addressing these challenges will pave the way for innovative training tools that improve patient outcomes.

## 5. Conclusions

Despite significant advancements in developing TMMs, achieving the ideal balance between durability, acoustic accuracy, and ease of fabrication remains challenging. PVCP-based phantoms are a promising solution due to their stability and cost-effectiveness. However, further validation and optimization are necessary to support their extensive implementation in clinical and training settings

## Figures and Tables

**Table 1:** Inclusion and exclusion criteria in detail.

Inclusion Criteria	Exclusion Criteria
Ultrasound phantom	Studies that use numerical or commercial breast phantoms
Phantoms made using tissue-mimicking materials	Studies published before 2013
Breast phantom	Studies written in languages other than English
Original research studies	Reviews, expert opinions, letters, conference proceedings, and book chapters
Studies written in English	
Studies published from 2013 to August 2023	

**Table 2:** An overview of tissue-mimicking materials (TMMs) used in breast ultrasound phantoms, detailing their speed of sound (SOS), acoustic attenuation coefficient, and elasticity across studies from 2013 to 2023.

Author (Year)	TMM	Phantom Type	SOS (m/s)	AC (dB/MHz/cm)	Elasticity (kPa)
Kashif et al., 2013 [18]	Silicone	Breast phantom	-	-	2–570
Vieira et al., 2013 [19]	Paraffin gel wax + glass microspheres	Breast phantom	1425.4–1480.3	0.32–2.04	-
Chun-Yen et al., 2014 [20]	Agarose + evaporated milk	Ultrasound-guided breast biopsy training	1480–1540	-	-
Manickam et al., 2015 [21]	Agar + N-propanol + graphite powder	Ultrasound and CT phantom	1564–1671	0.69–0.82	12.5–25
De Carvalho et al., 2016 [22]	PVCP + graphite powder	Ultrasound breast phantom	1379.3–1388	0.37–0.4	-
Jeong et al., 2016 [23]	PVCP + Al_2_O_3_	Ultrasound breast phantom	1370	0.71	-
Browne et al., 2017 [24]	IEC agar	Ultrasound breast phantom	1497–1553	0.6–2.0	-
Matheo et al., 2018 [25]	PVC plastisol + TiO_3_	Ultrasound breast phantom	1400	0.5	-
Ustbas et al., 2018 [26]	Silicone + PDMS	Ultrasound breast phantom	1290	12.99 dB/cm	-
Browne et al., 2019 [27]	IEC agar	Ultrasound breast phantom	1497–1553	0.6–2.0	-
Ng et al., 2019 [28]	IEC agar	Ultrasound breast and needle insertion feedback phantom	1479–1553	0.6–2	120–401
Suzuki et al., 2019 [29]	SEBS + paraffin oil	Ultrasound and CT phantom	1456–1503	0.4–1.2	-
Ruvio et al., 2020 [30]	IEC agar	Microwave, ultrasound, mammography, MRI, and CT phantom	1532–1710	0.73–4.0	-
Chatelin et al., 2020 [31]	PVCP + bis(2-ethylhexyl) adipate	MR and ultrasound elastography phantom	1400–1500	0.14–1.641	-
Altun et al., 2021 [32]	Gelatine + glycerol	Ultrasound breast phantom	-	-	-
Schmidt et al., 2021 [33]	Agar	Ultrasound breast and needle insertion feedback phantom	-	-	-
Browne et al., 2022 [34]	IEC agar	Ultrasound breast phantom	1551	0.46–0.6	-
Leonov et al., 2023 [35]	PVCP + graphite powder + metallic glitter	Ultrasound breast phantom	1400–1550	0.05–0.45	-
Hariyanto et al., 2023 [36]	PVC + DOP + graphite + silicone oil	Mammography and ultrasound phantom	1436–2021	0.51–0.063	-

## Data Availability

The original data presented in the study are openly available at DOI: 10.17632/bkr8dsp266.1.
